# In Silico and In Vitro Investigation of Apoptosis-Mediated Antiproliferative Activity of Capsaicin and Alpha-Lipoic Acid Against Prostate Cancer Cells

**DOI:** 10.3390/cimb48040376

**Published:** 2026-04-04

**Authors:** Ogunc Meral, Burcu Menekse Balkan, Dilek Nur Bestil, Serkan Sayiner, Deniz Ceylanli, Sare Uyurca, Sinem Pehlivan, Guzin Ozkurt, Gorkem Kismali, Tevhide Sel

**Affiliations:** 1 Department of Biochemistry, Faculty of Veterinary Medicine, Ankara University, Ankara 06070, Turkey; gorkemkismali@yahoo.com (G.K.); tevhidesel@gmail.com (T.S.); 2 Department of Biochemistry, Faculty of Veterinary Medicine, Burdur Mehmet Akif Ersoy University, Burdur 15200, Turkey; burcualpaslan@yahoo.com; 3 Department of Biochemistry, Institute of Health Science, Ankara University, Ankara 06070, Turkey; dileknurbbestil@gmail.com; 4 Department of Biochemistry, Faculty of Veterinary Medicine, Near East University, Nicosia 99138, North Cyprus, Mersin-10, Turkey; serkan.sayiner@neu.edu.tr (S.S.); deniz.ceylanli@neu.edu.tr (D.C.); 5 Department of Biochemistry, Faculty of Medicine, Duzce University, Duzce 81620, Turkey; sareuyurca@duzce.edu.tr; 6 Department of Medical Pharmacology, School of Medicine, Ankara Medipol University, Ankara 06050, Turkey; sinem.pehlivan@ankaramedipol.edu.tr; 7 Department of Biochemistry, Faculty of Veterinary Medicine, Aksaray University, Aksaray 68100, Turkey; guzinozkurt@aksaray.edu.tr

**Keywords:** alpha-lipoic acid, apoptosis, capsaicin, prostate cancer, survivin

## Abstract

Natural products are widely used in the treatment of cancer due to the side effects of chemotherapeutics, and a large number of natural compounds have been reported to have anticancer activities in different types of cancer. The aim of the study was to analyze the antiproliferative and apoptotic effects of capsaicin and alpha-lipoic acid on prostate cancer cells in silico and in vitro. The effects of capsaicin and alpha-lipoic acid on the proliferation of prostate cancer cells were assessed using MTT cell viability assays. Apoptotic protein levels were measured using Western blot analysis. Ligand–protein potential interactions of alpha-lipoic acid and capsaicin with survivin and bax proteins were examined using CB-Dock2 and SwissDock software. Capsaicin and alpha-lipoic acid significantly inhibited the proliferation of both prostate cancer cell lines in a dose- and time-dependent manner. Our data revealed that various concentrations of capsaicin, alpha-lipoic acid and their combinations caused remarkable downregulation of survivin expression on prostate cancer cells. According to the Vina scores, alpha-lipoic acid and capsaicin have the potential to interact strongly with survivin and bax proteins. We suggest that both natural compounds have the potential for treating prostate cancer according to the in vitro and in silico results.

## 1. Introduction

Prostate cancer is the second most common malignant cancer type among men worldwide. In some cases, it is highly aggressive and can spread rapidly to other tissues and organs, while in others it is slow-growing and best managed conservatively. Family history and older age are the most important risk factors for prostate cancer [1,2]. Despite recent advances in early diagnosis of the disease, it is asymptomatic in its early stages, leading to late diagnosis [3]. Even though radiation therapy and surgery have been an effective treatment strategy for the past ten years, prostate cancer remains one of the most dangerous types of cancer for men. Therefore, treatment challenges and the related side effects of chemotherapeutics have recently led researchers to seek natural alternatives in the treatment of prostate cancer [2,4].

Many studies have revealed that a large number of natural products exhibit potential anticancer effects and hence can play a significant role in new anticancer drug development. Natural products have shown promising results against various cancer types by modulating different signaling pathways, typically resulting in apoptosis [5,6]. Programmed cell death (apoptosis) plays a key role in several physiological states such as growth, tissue maintenance, organism homeostasis and the regulation of many pathophysiological conditions including cancer [7]. Apoptosis is essential for the precise body development and the removal of the redundant or damaged cells [8]. Many researchers have assumed that targeting specific molecules involved in apoptosis may offer the possibility of enhanced anticancer treatments [7].

Capsaicin (trans-8-methyl-N-vanillyl-6-noneamide) is a homovanillic acid derivative and the principal pungent component of chili peppers from the genus Capsicum. It has been demonstrated that capsaicin has several biomedical functions including anti-inflammatory, anti-obesity and antioxidant. Additionally, many studies have shown that capsaicin induces apoptosis in various cancer cell lines including pancreas, liver, lung and colonic, although not normal cells. Although the exact cellular mechanisms are not entirely understood [9], the role of capsaicin in carcinogenesis is debatable due to the conflicting study results. Many researchers have reported that capsaicin has chemopreventive and chemotherapeutic effects, while others have revealed it as a carcinogenic or a tumor promoter [10].

Alpha-lipoic acid (ALA), also known as thioctic acid (1,2-Dithiolane-3-pentanoic acid), is a natural product that is synthesized enzymatically in animals, humans and plants. ALA has been demonstrated to be a powerful antioxidant, playing an important role in scavenging reactive oxygen species (ROS) such as superoxide radicals, peroxyl radicals, and singlet oxygen. Many studies have revealed that ALA has beneficial effects on various diseases such as diabetes mellitus, Alzheimer’s disease and cancer [11,12]. Studies have stated that ALA triggers apoptosis and inhibits cell proliferation in various cancer cell lines such as colon and lung cancer. On the other hand, molecular and cellular mechanism underlying the apoptotic effect in cancer is not clearly understood [13].

Pharmacokinetic and toxicity analysis of molecules is a fundamental feature of the drug discovery program and has led to an increase in in silico studies related to this field. In silico studies are used for the preliminary evaluation of possible molecular interactions prior to in vitro and in vivo studies utilizing different databases, incorporating knowledge from toxicology, biostatistics, systems biology, computer science and many other related disciplines [14,15].

Molecular docking studies have been widely used for analyzing the interaction of a protein with a potential ligand. CB-Dock2 (http://cadd.labshare.cn/cb-dock2/) and SwissDock (https://www.swissdock.ch/) software are used to predict binding sites of ligands in these studies. Briefly, these applications rank the modes according to the Vina scores obtained from binding regions and provide 3D visualizations of the potential binding mode [16].

The aim of this study was to investigate the effect of capsaicin and ALA on proliferation and apoptosis, together with its association with apoptosis-related proteins survivin and bax, in PC-3 and DU-145 prostate cancer cell lines, both in silico and in vitro. Capsaicin and ALA were selected for a combination study based on their complementary, apoptosis-inducing mechanisms in cancer cells, suggesting potential for synergistic efficacy against prostate cancer cell lines. The apoptosis-mediated antiproliferative effects of these compounds on prostate cancer cells were investigated, and the interaction mechanisms of apoptosis-related proteins and compounds were investigated by molecular docking studies.

## 2. Materials and Methods

### 2.1. Reagents

Capsaicin was purchased from Calbiochem (San Diego, CA, USA). Stock solution was prepared in dimethyl sulfoxide (DMSO) and stored at −20 °C until required. Before the experiments, capsaicin stock solution was diluted in cell culture medium to final concentrations of 500, 250, 125, 62, 31 and 15 µM used in the wells. ALA was purchased from Sigma-Aldrich (St. Louis, MO, USA). Stock solution was prepared in DMSO and stored at −20 °C until required. Before the experiments, ALA stock solution was diluted in the medium of cell culture to final concentrations of 1000, 500, 250 and 125 µM respectively. Final concentration of DMSO was less than 0.1% in working solutions to avoid its cytotoxicity.

### 2.2. Cell Culture

Prostate cancer cell lines PC-3 (CRL-1435) and DU-145 (HTB-81) were purchased from American Type Culture Collection (Manassas, VA, USA). Cells were cultured in Dulbecco’s Modified Eagle Medium/F12 (DMEM/F12) media containing 10% fetal bovine serum (FBS) supplemented with 50 µg/mL gentamicin sulfate solution. Cells were grown in 25 cm^2^ or/and 75 cm^2^ vented cap flasks (BD Falcon) in a humidified 37 °C incubator with 5% CO_2_ atmosphere.

### 2.3. Cell Viability Assay

The effects of capsaicin and ALA on the cell proliferation of PC-3 and DU-145 prostate cancer cell lines were determined using the measurement of mitochondrial dehydrogenase activity using a MTT (3-(4,5-dimethylthiazol-2-yl)-2,5-diphenyltetrazolium bromide) cell viability assay. Cells were seeded in clear 96-well plates at a density of 5 × 10^3^ cells/well. After 24 h incubation, dead or unattached cells were removed by rinsing with phosphate-buffered saline (PBS). Cells were treated with various concentration of capsaicin alone (0, 15, 31, 62, 125, 250, 500 µM), ALA alone (0, 125, 250, 500, 1000 µM) and a combination of capsaicin (125 µM, 250 µM) and ALA (250, 500 and 1000 µM) for 24 and 48 h, respectively. After treatment, cells were washed with PBS, and 100 µL of MTT (5 mg/mL) was applied to each well. Following the 4 h incubation, the supernatant was removed and 100 µL of 10% sodium dodecyl sulfate (SDS) was added to each well to solubilize the formazan salt. Finally, absorbance was measured at 570 nm using microplate reader (Sunrise, Tecan, Salzburg, Austria). Cell viability was calculated as a percentage compared to untreated control cells. All experiments were performed in triplicate.

### 2.4. Western Blot Analysis

Prostate cancer cell lines were treated with capsaicin alone (0, 31, 62, 125 µM), ALA alone (0, 500 µM) and a combination of capsaicin (125 µM) and ALA (250, 500 and 1000 µM) for 24 h. After treatment, cells were washed with PBS, gently harvested using a cell scraper, and then washed 3 times with sterile PBS. Cells were lysed in a RIPA lysis buffer (Bio-Rad, Hercules, CA, USA) and total protein concentrations were measured using Pierce 660 nm protein assay reagent (Pierce/Thermo Scientific, Rockford, IL, USA). The protein expression of survivin and bax was determined using a semi-quantitative Western blot technique. Proteins were separated by 10% sodium dodecyl sulfate–polyacrylamide gel electrophoresis (SDS-PAGE) and equal amounts of proteins (20 µg) were applied to each well. Following electrophoresis, separated proteins were transferred onto nitrocellulose membranes. The membranes were blocked to prevent nonspecific bindings with 5% dry-milk in Tris-buffered saline containing Tween-20 (TBST) and then incubated with the corresponding primary antibodies at 4 °C overnight. Survivin (Cat. No: 2808, Cell Signalling Technology, Danvers, MA, USA, 1:1000 dilution) and bax (Cat. No: 2772, Cell Signalling Technology, USA, 1:1000 dilution) were used as a primary antibody and β-actin (Cat. No: 8457, Cell Signalling Technology, USA, 1:1000 dilution) was used as a loading control to normalize the levels of protein detected. After washing in TBST, membranes were incubated at room temperature for 2 h with biotin-xx anti-rabbit IgG secondary antibody (Cat. No: 2770, Invitrogen, Carlsbad, CA, USA, 1:5000 dilution) and signals were detected by streptavidin conjugate (Invitrogen, Carlsbad, CA, USA). Gel Logic 200 Imaging System (Kodak, New York, NY, USA) was used to scan the Western blot membranes.

### 2.5. Molecular Docking

The 3D structure of survivin and bax proteins (PDB ID: 1F3H; 4S0O) was obtained using the RSBC (Research Collaboratory for Structural Bioinformatics) PDB (Protein Data Bank) format. Protein structures were prepared according to the X-ray diffraction method and crystallographic water molecules were removed. The SMILE structures of ALA and capsaicin ligands were converted to SDF format using the ZINC20 molecular modeling package (CID: 864; 1548943). Molecular docking studies were performed using Cavity Detection-Guided Blind Docking (CB-Dock2) and SwissDock to determine the best conformational interaction of ALA and capsaicin with the identified target proteins survivin and bax [17,18].

### 2.6. Drug-likeness and Pharmacokinetic Analysis

The term ADME (Absorption, Distribution, Metabolism, Excretion and Pharmacokinetics), which constitutes the pharmacokinetics of the molecule, is crucial for drug development studies. In addition, Lipinski’s Rule of Five states that orally active compounds must consist of at least 4 criteria among partition coefficient (Log P), molecular weight (MW), number of hydrogen bond donors (HBD), number of hydrogen bond acceptors (HBA) and number of rotatable bonds. ALA and capsaicin compounds were examined using SwissADME (https://www.swissadme.ch/) and ADMETlab 2.0 (https://admetmesh.scbdd.com/) web servers for drug-likeness and pharmacokinetic properties [19].

### 2.7. Bioavailability Radar

A bioavailability radar of ALA and capsaicin was generated using the ADMETlab software program [19].

### 2.8. Statistical Analysis

All data analyses were performed with SPSS 22.0 software (SPSS Inc., Chicago, IL, USA). Statistical comparisons between control and treatment groups were performed using one-way ANOVA followed by Duncan’s multiple range test for post hoc comparisons. IC50 values were calculated by nonlinear regression analysis. *p* values < 0.05 were considered statistically significant. All experiments were performed in triplicate.

## 3. Results

### 3.1. Effects of Capsaicin and ALA on Cell Viability

**Figure 1:**
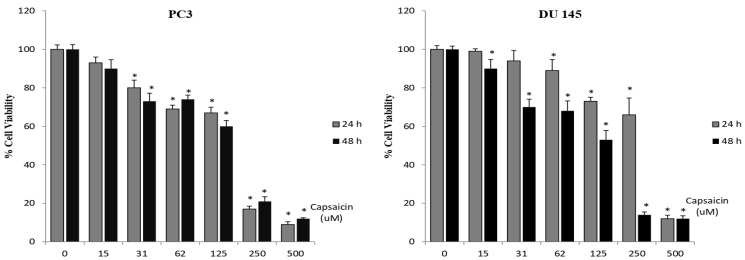
Effect of different concentration of capsaicin on cell viability. Two different prostate cancer cell lines (PC-3 and DU-145) were treated with different concentrations of capsaicin for 24 and 48 h. The viability of cells was determined by MTT cell viability assay and expressed as percentage of the control. Data are shown as mean ± SD; * *p* < 0.05 compared with the control group.

The effects of capsaicin and ALA on the cell viability of prostate cancer cell lines (PC-3, DU-145) were determined by MTT assay in vitro. Different concentrations of capsaicin and ALA were used to treat prostate cancer cell lines for 24 and 48 h. Capsaicin significantly suppressed the proliferation of both prostate cancer cell lines in a dose- and time-dependent manner (Figure 1). After 24 h of treatment, IC50 values of capsaicin were 201 µM in PC-3 cell line and 404 µM in DU-145 cell line and after 48 h of treatment; IC50 values of capsaicin were 199 µM in PC-3 cell line and 233 µM in DU-145 cell line, respectively. According to IC50 values, PC-3 cell line was more sensitive to capsaicin as compared with DU-145 cell line. In particular, significant inhibitory effects on cell viability for both cell lines following treatment with 125 and 250 µM concentrations of capsaicin were observed. In addition, capsaicin was found more effective when incubated for 48 h. These data indicate that capsaicin had potent antiproliferative effects against both prostate cancer cell lines.

**Figure 2:**
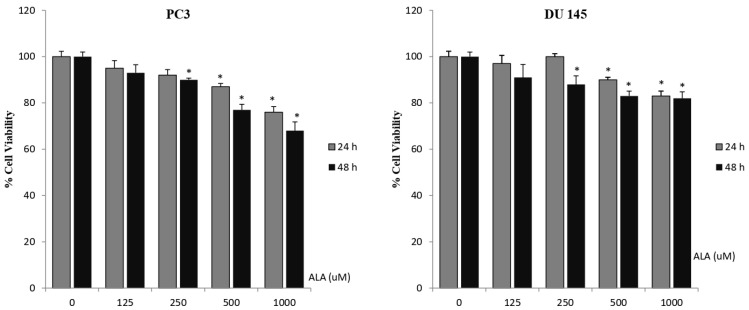
Effect of different concentrations of ALA on cell viability. Two different prostate cancer cell lines (PC-3 and DU-145) were treated with different concentrations of ALA for 24 and 48 h. The viability of cells was determined by MTT cell viability assay and expressed as percentage of the control. Data are shown as mean ± SD; * *p* < 0.05 compared with the control group.

Higher concentrations of ALA (250, 500, and 1000 µM) statistically significantly inhibited cell proliferation in both prostate cancer cell lines after 24 and 48 h of treatment. (Figure 2). After 24 h of treatment, IC50 values of ALA were 1880 µM in PC-3 cell line and 2040 µM in DU-145 cell line, and after 48 h of treatment, IC50 values of ALA were 1866 µM in PC-3 cell line and 1905 µM in DU-145 cell line, respectively. In particular, 48 h of treatment with the highest concentration of ALA (1000 µM) on PC-3 prostate cancer cell line statistically significantly decreased cell viability. ALA was found more effective after 48 h incubation. Our data indicate that even at the highest concentrations, ALA exhibited a mild effect on cell viability on both prostate cancer cell lines. We observed that capsaicin exhibited a greater antiproliferative effect than ALA at 125, 250 and 500 µM concentrations on both cell lines.

**Figure 3:**
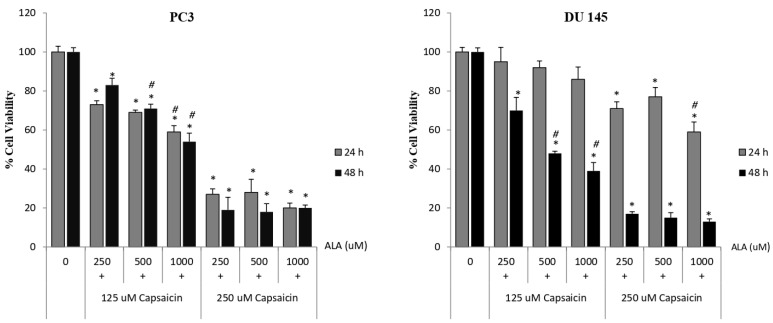
Effect of different concentrations of capsaicin and ALA combination on cell viability. Two different prostate cancer cell lines (PC-3 and DU-145) were treated with different concentrations of capsaicin and ALA for 24 and 48h. The viability of cells was determined by MTT cell viability assay and expressed as percentage of the control. Data are shown as mean ± SD; * < 0.05 compared with the control group; # < 0.05 compared with the lowest concentration of ALA (250 µM)-treated cells.

Different concentrations of ALA (250, 500, 1000 µM) in combination with 125 µM and 250 µM capsaicin were used to treat prostate cancer cell lines for 24 and 48 h individually to determine the effects of the combination. ALA and capsaicin combination significantly suppressed cell proliferation on both prostate cancer cell lines in a dose- and time-dependent manner (Figure 3). In addition, synergistic effects were not observed in any dose combinations of these compounds.

### 3.2. Effects of Capsaicin and ALA on Protein Expression

**Figure 4:**
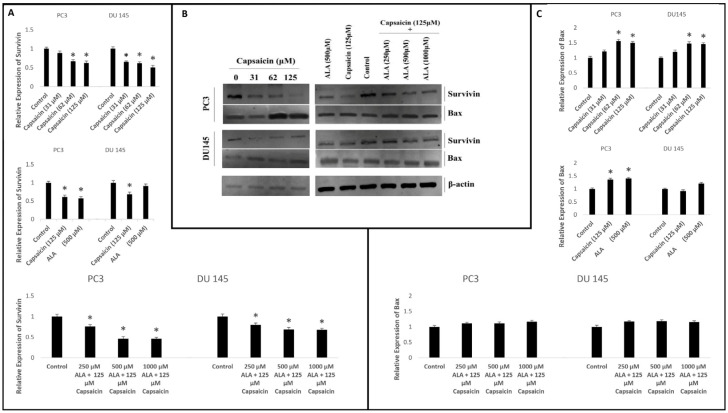
Effect of capsaicin, ALA and their combination on apoptosis-related protein expression in prostate cancer cells. Two different prostate cancer cell lines (PC-3 and DU-145) were treated with different concentration of capsaicin, ALA and their combination for 24 h. Survivin and bax protein expressions were determined using the Western blotting method (**B**). β-actin is used as a loading control. Quantitative analysis of survivin (**A**) and bax (**C**) protein. * *p* < 0.05 compared with the control group.

The effects of capsaicin and ALA on apoptosis were investigated using the Western blotting method. Different concentrations of capsaicin alone, ALA alone and their combinations were used to treat both prostate cancer cell lines for 24 h and the expression of apoptosis-related proteins survivin and bax was determined. Our data revealed that treatment with 62 and 125 µM capsaicin significantly downregulated survivin protein expression for both prostate cancer cell lines compared with the levels in the control group (Figure 4A). Also, bax protein levels were found to be upregulated in the same capsaicin concentrations on both prostate cancer cell lines. Additionally, our data showed that treatment of PC-3 prostate cancer cell line with 500 µM ALA downregulated survivin protein expression while upregulating bax protein expression in comparison with the control group (Figure 4B). However, no remarkable change in survivin and bax protein expression was observed on DU-145 cell line treated with 500 µM ALA. Our data showed that the ALA and capsaicin combination downregulated survivin protein expression on both prostate cancer cell lines in a dose-dependent manner (Figure 4C). On the other hand, no remarkable change in bax protein expression was observed for both prostate cancer cell lines treated with the capsaicin and ALA combination in a dose-dependent manner. Therefore, these data indicate that capsaicin alone, ALA alone and their combination caused remarkable downregulation of survivin expression on prostate cancer cell lines.

### 3.3. Molecular Docking Analysis

**Figure 5:**
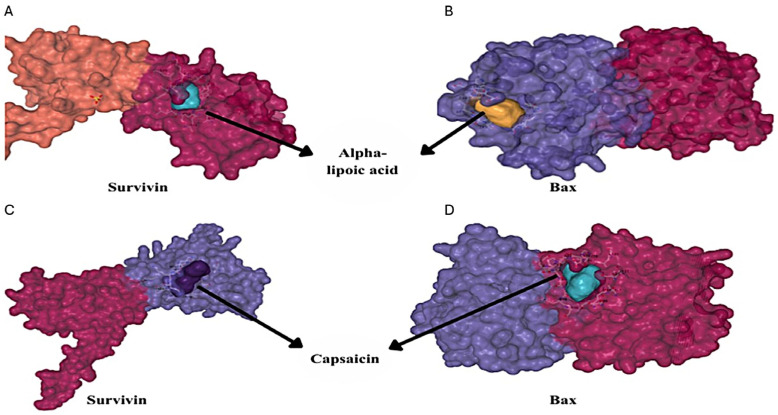
Molecular docking images of survivin and ALA (**A**), bax and ALA (**B**), survivin and capsaicin (**C**), and bax and capsaicin (**D**).

To investigate the potential for molecular interactions, in silico binding of ALA and capsaicin to survivin and bax proteins was performed using CB-Dock2 and SwissDock programs. The resulting Vina scores are presented in Table 1, and possible binding images are shown in Figure 5. The data were obtained to express the optimum Vina score, gap size, RMSD, binding coordinates and the chain of hydrophobic residues of the protein. Both capsaicin and ALA interacted with survivin and bax proteins according to the Vina scores (bax–capsaicin ≥ survivin–capsaicin ≥ surviving–ALA ≥ bax–ALA). Molecular docking studies revealed that capsaicin potentially binds to survivin at amino acid residues Phe13-Leu14 and to bax at residues Leu27-Gln28. Similarly, ALA potentially binds to survivin at amino acid residues Pro12-Phe13 and to bax at residues Gln28-Ile31. Capsaicin formed critical interactions on survivin and bax with favorable binding energies of −6.6 and −6.7 kcal/mol, respectively. In comparison, ALA showed weaker binding energies (−4.9 kcal/mol for survivin and −4.7 kcal/mol for bax), suggesting that ALA may have a more limited direct interaction with these apoptotic regulators.

### 3.4. Drug-likeness and Pharmacokinetic Properties

The pharmacokinetic profiles of ALA and capsaicin were analyzed using SwissADME and ADMETlab web servers. The ADME servers enabled the physicochemical properties of the compounds to be examined, including octanol/water partition coefficient (log Po/w), molecular weight and relative absorption in the intestine (Table 2). ALA and capsaicin appear to meet most of the drug candidate rules, but capsaicin does not meet the Pfizer rule (Table 3).

### 3.5. Bioavailability Radar

**Figure 6:**
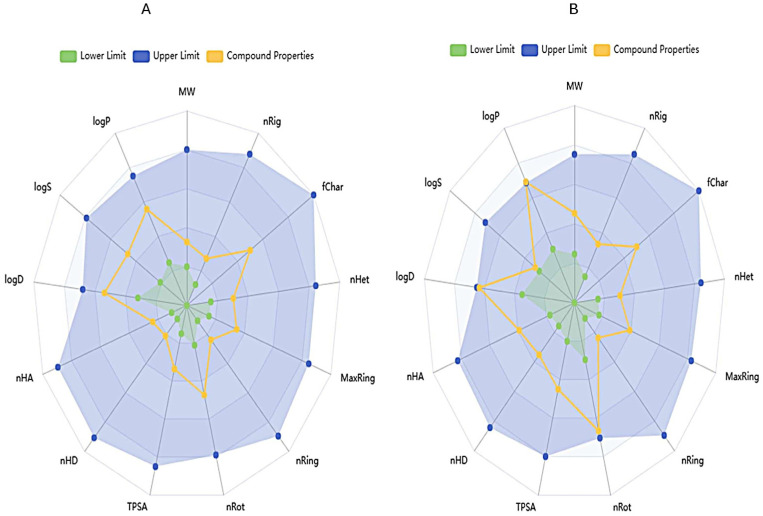
Bioavailability radar of ALA (**A**) and capsaicin (**B**) (MW: molecular weight; nRig: number of rigid bonds; fChar: fraction of charged atoms; nHet: number of heteroatoms; MaxRing: maximum ring size; nRing: number of rings; nROT: number of rotatable bonds; TPSA: topological polar surface area; nHD: number of hydrogen bond donors; nHA: number of hydrogen bond acceptors; logD: logarithm of the distribution coefficient; logS: logarithm of (aqueous) solubility; logP: logarithm of the partition coefficient).

The parameters on the bioavailability radar are: molecular weight, number of rigid bonds, fraction of charged atoms, number of heteroatoms, maximum ring size, number of rings, number of rotatable bonds, topological polar surface area, number of hydrogen bond donors, number of hydrogen bond acceptors, logarithm of the distribution coefficient, logarithm of (aqueous) solubility and logarithm of the partition coefficient. ALA (Figure 6A) and capsaicin (Figure 6B) are predicted to be orally bioavailable as the parameters do not fall outside the blue area.

## 4. Discussion

Prostate cancer remains one of the most dangerous cancers among men due to treatment challenges and side effects of chemotherapeutics. The sustained increase in prostate cancer incidence, challenges in conventional therapies, and high toxicity of chemotherapeutics have led to alternative treatment options in recent years. Natural compounds, such as capsaicin, are considered potential anticancer agents as they have an enormous suppressive effect on various cancer cells [2,20].

Our results showed that capsaicin significantly inhibited cell viability of the PC-3 and DU-145 cell line. The PC-3 cell line was more sensitive to capsaicin when compared with DU-145 cell line. Sánchez et al. [21] reported that capsaicin dose-dependently inhibited cell viability, with an effective dose of 40 µM in LNCaP cell line and 20 μM in PC3 cell line. Zhu et al. [22] also demonstrated that capsaicin downregulated prostate cancer stem cell markers and inhibited the growth of PC-3 and DU145 prostate cancer stem cells. Lewinska et al. [23] revealed that elevated doses of capsaicin (≥100 μM) led to decreased cell proliferation of DU-145 cancer cell line. In the present study, we observed that capsaicin has a significant inhibitory effect of cell viability on both cell lines after treatment with 125 and 250 µM concentrations. The antiproliferative effect of capsaicin on different types of cancer such as hepatocellular carcinoma [24], osteosarcoma [25], colorectal cancer [26] and pancreatic cancer [27] has been reported and our findings indicate that capsaicin also has a potent antiproliferative effect against both prostate cancer cell lines.

ALA, which is a naturally occurring antioxidant dithiol compound, has shown promising antiproliferative and anticancer effects on different types of cancer cell lines. Studies demonstrated that ALA effectively reduced the cell growth of LNCaP and DU-145 prostate cancer lines [28]. Our results demonstrate that ALA treatment on both prostate cancer cell lines significantly inhibited cell proliferation only at high concentrations (>250 µM). Studies have indicated that ALA has antiproliferative effects on different cancer cells such as hepatocellular carcinoma [29] and breast cancer [30]. Our findings indicated that ALA showed limited effect on cell viability on both prostate cancer cell lines. However, capsaicin–ALA combination showed a greater antiproliferative effect than ALA alone but similar to capsaicin alone, with no evidence of synergy; we suggest that these results are associated with the antiproliferative effect of capsaicin even at lower doses.

Apoptosis plays crucial role in cancer development and disturbance of this process is associated with carcinogenesis. Thus, induction of apoptosis in cancer cells has recently become a rational therapeutic strategy [31]. It has been shown that capsaicin induces apoptosis and inhibits cell proliferation in various cancer cells in vivo and in vitro [32,33,34]. Sánchez et al. [35] reported that treatment of PC-3 prostate cancer cell line with capsaicin resulted in an induction of apoptosis via activation of caspase-3, generation of the reactive oxygen species and dissipation of the mitochondrial inner transmembrane potential. Also, it has been shown that capsaicin promotes cell death by activating AMP-activated kinase (AMPK) in LKB1-expressing LNCaP and PC3 prostate cancer cells but not in DU-145 cell line [36].

Our data indicate that capsaicin treatment is associated with downregulation of survivin protein expressions and upregulation of bax levels on both prostate cancer cell lines. Additionally, capsaicin potentially interacted with survivin and bax proteins according to the Vina scores. Apoptosis inhibitor protein survivin is highly expressed in various cancer cells and exerts its anti-apoptotic function by inhibiting the activation of effector caspases. Furthermore, survivin levels are a crucial prognostic marker in a large number of malignancies. The association between survivin expression and progression and aggressiveness of prostate cancer has been well established [37].

Another apoptosis-related protein bax is a pro-apoptotic member of the Bcl-2 protein family and has an important role in the regulation of programmed cell death [38]. Chen et al. [39] reported that capsaicin inhibited proliferation and induced apoptosis in breast cancer both in vivo and in vitro via downregulation of FBI-1, Ki-67, Bcl-2, and survivin and upregulation of bax protein levels. Additionally, Zhang et al. [27] found that capsaicin inhibited AsPC-1 and BxPC-3 pancreatic cancer cell proliferation and induced apoptosis via upregulation of bax expression, downregulation of Bcl-2 and survivin proteins, and a significant release of cytochrome c and AIF to the cytosol. They proposed that these findings were linked to the generation of reactive oxygen species and the sustained disruption of the mitochondrial membrane potential. In this research, we observed that capsaicin significantly downregulated the expression of survivin and upregulated the expression of pro-apoptotic protein bax for both prostate cancer cell lines. These findings may be related to the generation of reactive oxygen species. We therefore assume that these findings are very important since it has been shown that survivin downregulation could be effective in sensitizing prostate cancer cells to chemotherapeutic agents both in vivo and in vitro [40].

The use of natural products such as ALA in alternative medicine as therapeutic agents has increased tremendously. ALA has been used in the management of diseases like breast cancer and is known for its protective effects against cancer. It has also been stated that ALA induces cell apoptosis and inhibits cell proliferation in cancer cells by reducing oxidative stress accumulated by cancer cells [41,42]. Simbula et al. [29] showed that ALA inhibited hepatocellular carcinoma cell growth and induced apoptosis via survivin downregulation, bax upregulation and activation of caspase-3. Also, studies have shown that the treatment of breast cancer cells with ALA led to downregulation of Bcl-2 and upregulation of bax protein and induced cell apoptosis [30].

The results of our study indicate that treatment of PC-3 prostate cancer cell line with 500 µM ALA led to downregulation of survivin protein expression and upregulation of bax protein expression. Also, both ALA and capsaicin formed stable complexes with apoptotic proteins, indicating potential binding interactions based on the calculated Vina scores. We acknowledge that the observed changes in protein expression do not conclusively demonstrate direct binding between the compounds and their targets. Such effects may also result from indirect regulation. This potential binding is consistent with our other findings and supports the role of survivin in mediating apoptosis in prostate cancer cells. Moreover, our data showed that the ALA and capsaicin combination downregulated survivin protein expressions on both prostate cancer cell lines in a dose-dependent manner. Although combinations of capsaicin and ALA significantly suppressed cell proliferation in a dose- and time-dependent manner, no statistically significant synergistic interaction was detected at any tested dose; this lack of synergy is likely due to the potent, multitargeted efficacy of capsaicin as a monotherapy, or may be due to them targeting the same general pathway. Taken together, these findings demonstrate that treatment of ALA alone or combination with capsaicin induced apoptosis and these data may be related to reduction in oxidative stress accumulated by prostate cancer cells [42].

## 5. Conclusions

In conclusion, our present study demonstrated that capsaicin and ALA mediate inhibition of cell proliferation and induce cell apoptosis in prostate cancer cell lines. The interaction mechanisms between capsaicin and ALA with survivin protein were investigated via molecular docking analysis. Our results indicated that the molecular mechanism of cell apoptosis is related to the downregulation of survivin protein by both natural compounds in prostate cancer cells. According to the pharmacokinetic, molecular docking and in vitro antiproliferative and apoptotic analyses, we suggest that capsaicin and ALA have the potential to be used in the prevention of prostate cancer and targeting survivin protein with these compounds may provide a good alternative strategy for prostate cancer treatment. A limitation of this study is that we only used androgen-independent prostate cancer cell lines (PC-3 and DU145) and did not include an androgen-dependent cell line like LNCaP; therefore, our findings may not apply to hormone-sensitive prostate cancer. Future research should progress to animal models, particularly xenograft models, to conduct in vivo validation and assess the therapeutic efficacy and systemic safety of capsaicin and ALA within a complex physiological environment.

## Figures and Tables

**Table 1:** Molecular docking analysis of capsaicin and ALA ligands with survivin and bax proteins.

Protein	Ligand	Vina Score (kcal/mol)	Cavity Volume (Å^3^)	Pocket RMSD	Center (x, y, z)	Docking Size (x, y, z)	Contact Residues
Survivin	Capsaicin	−6.6	226	0.88	57, −17, 35	24, 24, 24	Chain A: PHE13 LEU14 LYS15 ARG18 ALA39 GLU40 ALA41 PHE58 PHE59 ILE74 HIS77 LYS78 ALA85 PHE86 LEU87 SER88 VAL89 LYS90 LYS91 GLN92 PHE93 LEU96 PHE101 LEU104
Survivin	ALA	−4.9	565	1.20	29,1, 17	18, 18, 18	Chain A: PHE13 LEU14 LYS15 ASP16 ARG18 ALA39 GLU40 ALA41 PHE58 ILE74 HIS77 LYS78 PHE86 LEU87 VAL89 LYS90 LYS91 GLN92 PHE93 GLU94 LEU96 PHE101 LEU104
Bax	Capsaicin	−6.7	344	0.92	17, −9, 14	24, 24, 24	Chain A: GLN28 ILE31 GLN32 ALA35 GLU41 ALA42 PRO43 LEU45 ALA46 LEU47 ASP48 PRO49 VAL121 LEU125 PRO130 GLU131 ILE133 ARG134 MET137
Bax	ALA	−4.7	306	1.55	49, 9, 19	18, 18, 18	Chain B: GLN28 ILE31 GLN32 ALA35 GLU41 ALA42 PRO43 LEU45 ALA46 LEU47 LEU125 PRO130 GLU131 ILE133 ARG134 MET137

**Table 2:** Pharmacokinetic properties of ligands.

Ligand	Molecular Formula	Molecular Weight (g/mol)	Rotatable Bonds	H Bond Acceptors	H Bond Donors	LogP_O/W_	GI Absorption	Water Solubility
ALA	C_8_H_14_O_2_S_2_	206.3	5.0	2.0	1.0	1.851	LOW	SOLUBLE
Capsaicin	C_18_H_27_NO_3_	305.4	10.0	4.0	2.0	3.041	LOW	LOW

**Table 3:** Properties of ligands as drug candidates.

Ligand	Lipinski Rule	Pfizer Rule	GSK Rule	Golden Triangle
ALA	Accepted	Accepted	Accepted	Accepted
Capsaicin	Accepted	Rejected	Accepted	Accepted

## Data Availability

The data presented in this study are available on request from the corresponding author.
